# Loss of phosphatidylserine flippase β-subunit *Tmem30a* in podocytes leads to albuminuria and glomerulosclerosis

**DOI:** 10.1242/dmm.048777

**Published:** 2021-06-25

**Authors:** Wenjing Liu, Lei Peng, Wanli Tian, Yi Li, Ping Zhang, Kuanxiang Sun, Yeming Yang, Xiao Li, Guisen Li, Xianjun Zhu

**Affiliations:** 1 Health Management Center, Sichuan Provincial People's Hospital, School of Medicine, University of Electronic Science and Technology of China, Chengdu, Sichuan 610072, China; 2 The Sichuan Provincial Key Laboratory for Human Disease Gene Study, Center for Medical Genetics, Sichuan Provincial People's Hospital, University of Electronic Science and Technology of China, Chengdu, Sichuan 610072, China; 3 Department of Nephrology, Sichuan Academy of Medical Sciences and Sichuan Provincial People's Hospital, Chengdu, Sichuan Clinical Research Center for Kidney Diseases, Sichuan 610072, China; 4 Key Laboratory of Tibetan Medicine Research, Chinese Academy of Sciences and Qinghai Provincial Key Laboratory of Tibetan Medicine Research, Northwest Institute of Plateau Biology, Xining, Qinghai 810008, China; 5 Research Unit for Blindness Prevention of the Chinese Academy of Medical Sciences (2019RU026), Sichuan Academy of Medical Sciences and Sichuan Provincial People's Hospital, Chengdu, Sichuan 610072, China; 6 Department of Ophthalmology, Shangqiu First People's Hospital, Shangqiu, Henan 476000, China; 7 Natural Products Research Center, Institute of Chengdu Biology, Sichuan Translational Medicine Hospital, Chinese Academy of Sciences, Chengdu, Sichuan 610072, China

**Keywords:** *Tmem30a*, Albuminuria, Focal segmental glomerulosclerosis, Glomerulosclerosis

## Abstract

The asymmetric distribution of phosphatidylserine (PS) in the cytoplasmic leaflet of eukaryotic cell plasma membranes is regulated by a group of P4-ATPases (named PS flippases) and the β-subunit TMEM30A. Podocytes in the glomerulus form a filtration barrier to prevent the traversing of large cellular elements and macromolecules from the blood into the urinary space. Damage to podocytes can disrupt the filtration barrier and lead to proteinuria and podocytopathy. We observed reduced TMEM30A expression in patients with minimal change disease and membranous nephropathy, indicating potential roles of TMEM30A in podocytopathy. To investigate the role of *Tmem30a* in the kidney, we generated a podocyte-specific *Tmem30a* knockout (KO) mouse model using the NPHS2-Cre line. *Tmem30a* KO mice displayed albuminuria, podocyte degeneration, mesangial cell proliferation with prominent extracellular matrix accumulation and eventual progression to focal segmental glomerulosclerosis. Our data demonstrate a critical role of *Tmem30a* in maintaining podocyte survival and glomerular filtration barrier integrity. Understanding the dynamic regulation of the PS distribution in the glomerulus provides a unique perspective to pinpointing the mechanism of podocyte damage and potential therapeutic targets.

## Abstract (precis)

**Summary:** Using analyses of podocyte-specific *Tmem30a* knockout mice and TMEM30A expression in patients with podocytopathy, we demonstrate a critical role of *Tmem30a* in maintaining podocyte survival and glomerular filtration barrier integrity.

## INTRODUCTION

Phosphatidylserine (PS) is asymmetrically and dynamically distributed across the lipid bilayer in eukaryotic cell membranes (Devaux, 1991). Such dynamic distribution is preserved by flippases, one of the most important P4-ATPases, which possess flippase activity that catalyses lipid transportation from the outer to the inner leaflet to generate and maintain phospholipid asymmetry (Daleke and Lyles, 2000). The PS asymmetry maintained by P4-ATPases is essential to various cellular physiological and biochemical processes, including vascular trafficking, cell polarity and migration, cell apoptosis and cell signalling events (Daleke and Lyles, 2000; Dhar et al., 2004; van der Mark et al., 2014; Liu et al., 2017; Mansergh et al., 2005).

As the β-subunit of P4-ATPases (except ATP9A and ATP9B), TMEM30 family proteins play essential roles in the proper folding and subcellular localization of P4-ATPases (van der Velden et al., 2010; Folmer et al., 2012). The TMEM30 (also called CDC50) family includes TMEM30A, TMEM30B and TMEM30C, of which TMEM30A interacts with 11 of the 14 P4-ATPases (Bryde et al., 2010; Takatsu et al., 2011, 2016; Coleman et al., 2009, 2014). Our previous studies have demonstrated that TMEM30A deficiency causes a series of disorders: retarded retinal angiogenesis, Purkinje cell, retinal bipolar cell and photoreceptor cell degeneration, impaired foetal liver erythropoiesis, intrahepatic cholestasis and chronic myeloid leukaemia (Liu et al., 2017; Zhang et al., 2019; Yang et al., 2018, 2019a, 2019b; Zhang et al., 2017; Li et al., 2018).

The glomerular filtration barrier includes three layers: fenestrated endothelial cells, the glomerular basement membrane (GBM) and glomerular epithelial cells (podocytes). Podocytes consist of a cell body that gives rise to major processes and minor foot processes (FPs). The FPs of neighbouring podocytes form a branched interdigitating network, and the space between adjacent FPs is covered by a multiprotein complex called the slit diaphragm (SD), the final barrier (Tryggvason et al., 2006). The glomerular filtration barrier prevents the traversing of large cellular elements and macromolecules from the blood into the urinary space, and defects in the selective barrier result in albuminuria and nephrotic syndrome. Damage to podocytes can disrupt the filtration barrier, which is a key step in proteinuria and podocytopathy [including focal segmental glomerulosclerosis (FSGS), minimal change disease (MCD), membranous nephropathy (MN) and diabetic nephropathy (DN)], as well as other types of kidney disease [such as immunoglobin A nephropathy (IgAN) and lupus nephritis]. FSGS is one of the most widely used disease models to study podocytopathy and proteinuria (Bose and Cattran, 2014).

We observed markedly diminished TMEM30A expression in patients with MCD and MN, and also a downward trend of its expression in DN, suggesting that TMEM30A may have important functions in the kidney. Given that *Tmem30a* is essential for tissues with high TMEM30A expression, such as retina, cerebellar and hepatic tissue, and that *Tmem30a* is highly expressed in the kidney, we set out to elucidate the role of *Tmem30a* in the kidney by generating a podocyte-specific *Tmem30a* knockout (KO) model. *Tmem30a* KO mice displayed albuminuria, podocyte injury and loss, mesangial cell proliferation with prominent extracellular matrix (ECM) accumulation and eventual progression to FSGS. Taken together, our findings demonstrate that *Tmem30a* plays a critical role in maintaining podocyte survival and glomerular filtration barrier integrity.

## RESULTS

### TMEM30A expression is reduced in patients with podocytopathy, including MCD and MN

**Fig. 1.**
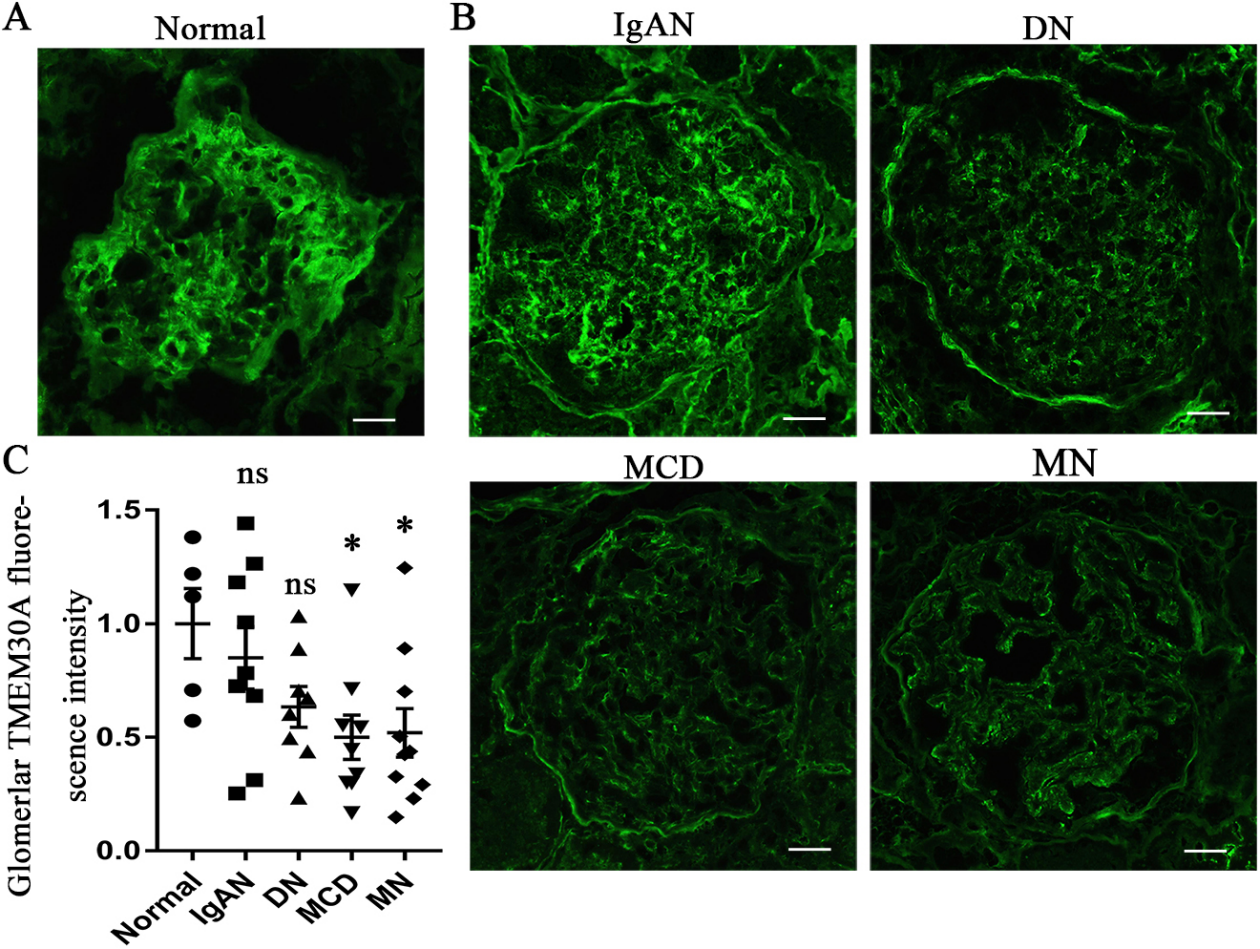
**Immunofluorescence staining of human glomeruli revealed reduced expression of TMEM30A in MCD and MN patients.** (A) Immunofluorescence images of TMEM30A expression in normal human glomerular tissue. (B) Immunofluorescence images of TMEM30A expression in glomerular tissue from human patients with IgAN, DN, MCD and MN. (C). Quantification of the intensity of fluorescent staining for human glomerular TMEM30A. Values represent the mean±s.e.m. ns, not significant; *P<0.05 versus normal group, by one-way ANOVA. Normal group (*n*=5) versus IgAN group (*n*=9), *P*=0.455; normal group versus DN group (*n*=8), *P*=0.079; normal group versus MCD group (*n*=9), *P*=0.043; normal group versus MN group (*n*=10), *P*=0.019. IgAN, immunoglobulin A nephropathy; DN, diabetic nephropathy; MCD, minimal change disease; MN, membranous nephropathy. Scale bars: 50 μm.

TMEM30A is expressed in human glomeruli (Fig. 1A). To evaluate the clinical importance of TMEM30A, we analysed the expression of TMEM30A in kidney samples from patients with podocytopathy (MCD, MN and DN), samples from patients with IgAN and adjacent normal tissues from patients with renal tumours as controls (clinical information is provided in Table 1). We observed significantly reduced TMEM30A expression in the glomeruli of patients with MCD or MN compared with that in the controls (Fig. 1B,C). Conversely, the TMEM30A expression level in glomeruli from IgAN patients showed no significant reduction compared with that in the controls. Although the expression of TMEM30A in glomeruli from DN patients showed a downward trend compared with that in the controls, this difference was not significant. These data suggest that the expression of *TMEM30A* is decreased in podocytopathy, especially in MCD and MN, and that TMEM30A could be essential for podocytes.

**Table 1.**
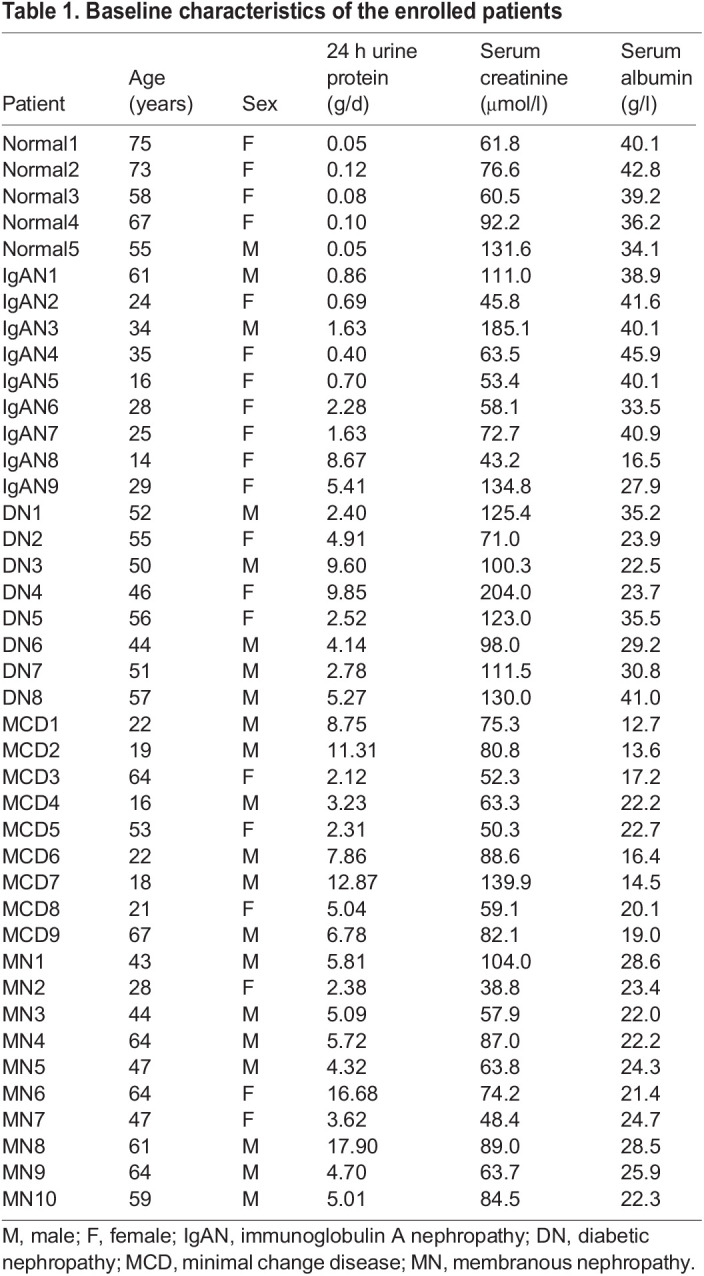
Baseline characteristics of the enrolled patients

### Generation of podocyte-specific *Tmem30a* KO mice

**Fig. 2.**
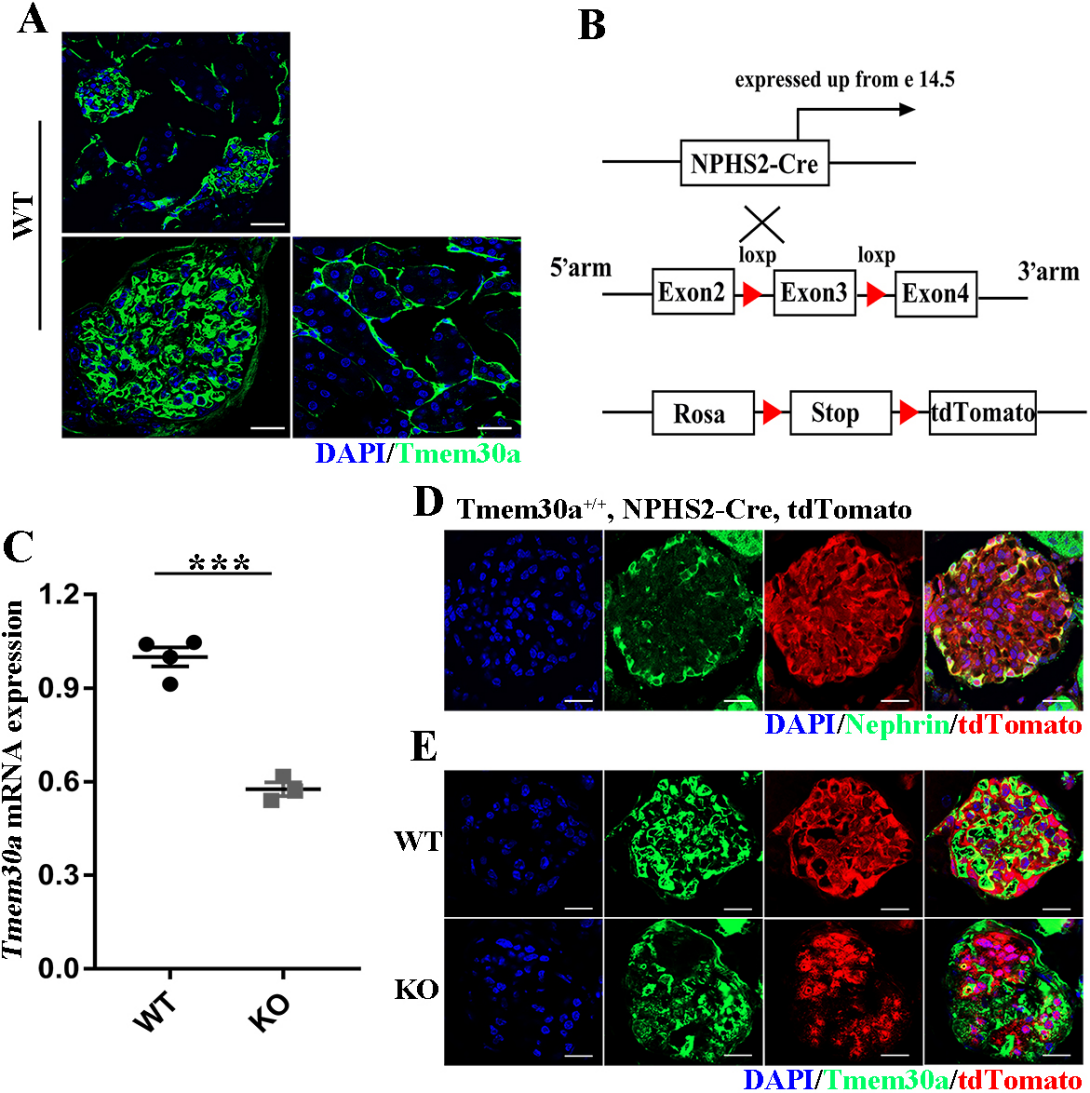
**Generation of podocyte-specific *Tmem30a* conditional knockout (KO) mice.** (A) Cryosections of the kidney from 5-month-old wild-type (WT) mice were immunostained with anti-TMEM30A antibody (green). The upper panel shows a lower-magnification TMEM30A staining image of the glomerular cortex, and the lower panels show high-resolution TMEM30A immunostaining in the glomeruli and renal tubules, respectively. *Tmem30a* is highly expressed in the glomeruli. (B) Schematic showing the targeting strategy for generating podocyte-specific *Tmem30a* KO mice. Rosa-tdTomato reporter mice were used to monitor Cre expression. (C) Quantitative PCR showed the relative mRNA expression of *Tmem30a* in the glomeruli of KO mice compared with that in WT mice. *n*=4. Values represent the mean±s.e.m. ***P<0.001, by unpaired Student's *t*-test. (D) The ROSA-tdTomato reporter was introduced to monitor the expression of Cre recombinase (red). Podocytes were labelled with the podocyte-specific marker nephrin (green). TdTomato-expressing cells were colocalized with nephrin-labelled podocytes, indicating the specific expression of NPHS2-Cre. (E) Localization of TMEM30A and Rosa-tdTomato in WT and KO mice by immunofluorescence, suggesting that *Tmem30a* was knocked out in podocytes. Scale bars: 25 μm (A, upper panel, D,E); 10 μm (A, lower panels).

Previous studies have demonstrated the essential role of *Tmem30a* in several vital tissues. In the retina, *Tmem30a* is important for the survival of retinal photoreceptor and rod bipolar cells (Yang et al., 2019a; Zhang et al., 2017). In the cerebellum, *Tmem30a* loss results in early-onset ataxia and cerebellar atrophy (Yang et al., 2018). In the liver, *Tmem30a* deficiency impairs mouse foetal liver erythropoiesis, causes intrahepatic cholestasis by affecting the normal expression and localization of bile salt transporters, and causes intrahepatic cholestasis (Liu et al., 2017; Yang et al., 2019b). In the haematopoietic system, *Tmem30a* is critical for the survival of haematopoietic cells and leukocytes (Li et al., 2018). *Tmem30a* is expressed in the retina, brain, cerebellum, liver, heart, kidney, spine and testis (Folmer et al., 2012; Zhang et al., 2017; Stevens and Oltean, 2018), but its role in the kidney remains unknown. To define the role of *Tmem30a* in the kidney, we first assessed the expression of *Tmem30a* in mouse kidney sections by immunostaining with a proven anti-TMEM30A antibody (Zhang et al., 2017). Kidney cryosections were immunostained with specific antibodies against *Tmem30a* (Fig. 2A). *Tmem30a* is highly expressed in the glomeruli, which implies a vital role of *Tmem30a* in glomerular filtration. To investigate this role of *Tmem30a*, we generated podocyte-specific *Tmem30a* KO *Tmem30a^loxP/loxP^*; NPHS2-Cre (hereafter named *Tmem30a* KO) mice by crossing *Tmem30a^loxP/loxP^* with podocin-Cre NPHS2-Cre mice (Fig. 2B). *Tmem30a* expression was reduced by ∼55% in the glomeruli of *Tmem30a* KO mice compared with that in the control mice (Fig. 2C). Given the presence of Cre only in the podocytes, the deletion efficiency was fairly good. ROSA26-tdTomato was used to verify the specific expression of podocin-Cre in podocytes. We crossed *Tmem30a*^loxP/+^; NPHS2-Cre; Rosa-tdTomato mice with *Tmem30a^loxP/loxP^* mice to generate littermate *Tmem30a*^+/+^; NPHS2-Cre; Rosa-tdTomato and *Tmem30a^loxP/loxP^*; NPHS2-Cre; Rosa-tdTomato mice to evaluate the KO specificity of *Tmem30a* in podocytes (Fig. 2B-E). In summary, these data demonstrate the successful elimination of *Tmem30a* in *Tmem30a^loxP/loxP^*; NPHS2-Cre mice.

### Podocyte-specific deletion of *Tmem30a* results in albuminuria

**Fig. 3.**
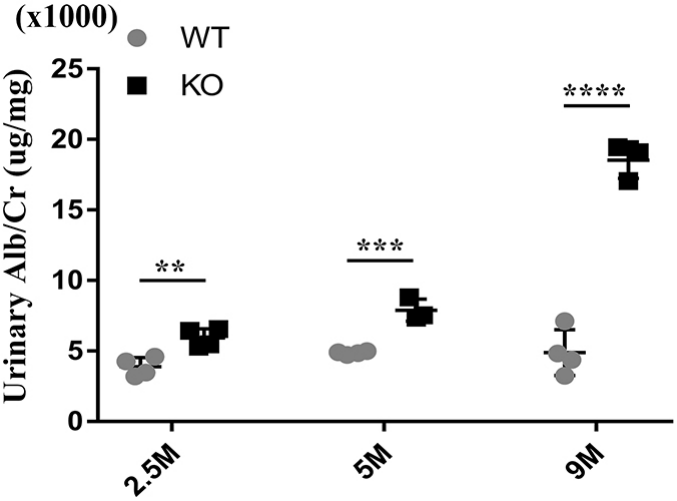
**Deletion of *Tmem30a* in podocytes resulted in albuminuria.** Urine biochemical analysis was performed in 2.5-, 5- and 9-month-old WT and *Tmem30a* KO mice. Quantitation of urinary albumin in WT and *Tmem30a* KO mice showed that *Tmem30a* KO mice exhibited albuminuria at 2.5 months of age, which became severe by 5 and 9 months of age. *n*=3 for both WT and KO mice. Values represent the mean±s.e.m. **P<0.01, ***P<0.001, ****P<0.0001, by unpaired Student's *t*-test.

*Tmem30a* KO mice were born at a ratio that is consistent with classic Mendelian segregation. No obvious morphological abnormalities were observed in *Tmem30a* KO mice upon gross examination. Although they appeared to be normal in terms of body size, the albuminuria level in *Tmem30a* KO mice increased significantly compared with that in control mice from 2.5 months after birth (Fig. 3). By the fifth and ninth months after birth, the albuminuria level continued to rise, indicating sustained impairment of the glomeruli and selective barrier (Fig. 3). To rule out sex differences, we measured albuminuria levels in female and male mice at the same time point (8 months), and the results showed three times increased albuminuria levels in both male and female *Tmem30a* KO mice compared with wild-type (WT) mice of the same sex (Fig. S1), suggesting that the albuminuria phenotype was similar in male and female *Tmem30a* KO mice. All subsequent experiments were performed using male mice.

Albuminuria is an unambiguous symptom of the compromised integrity of the glomerular filtration barrier (Coleman and Molday, 2011). With increased protein passage from blood into urine, the proximal tubular reuptake mechanism is stimulated to reabsorb an increasing amount of protein until the reabsorption capacity is saturated (Remuzzi et al., 2002). Once the amount of protein excreted from blood exceeds the reabsorption capacity of the proximal tubule, albuminuria occurs. Mounting evidence indicates that albuminuria is one of the major features of various kidney diseases, or at least that albuminuria accelerates kidney disease progression to end-stage renal failure (Maunsbach, 1966). This indicates that defects in *Tmem30a* are a crucial cause of albuminuria.

### *Tmem30a* is essential for the survival and function of podocytes

**Fig. 4.**
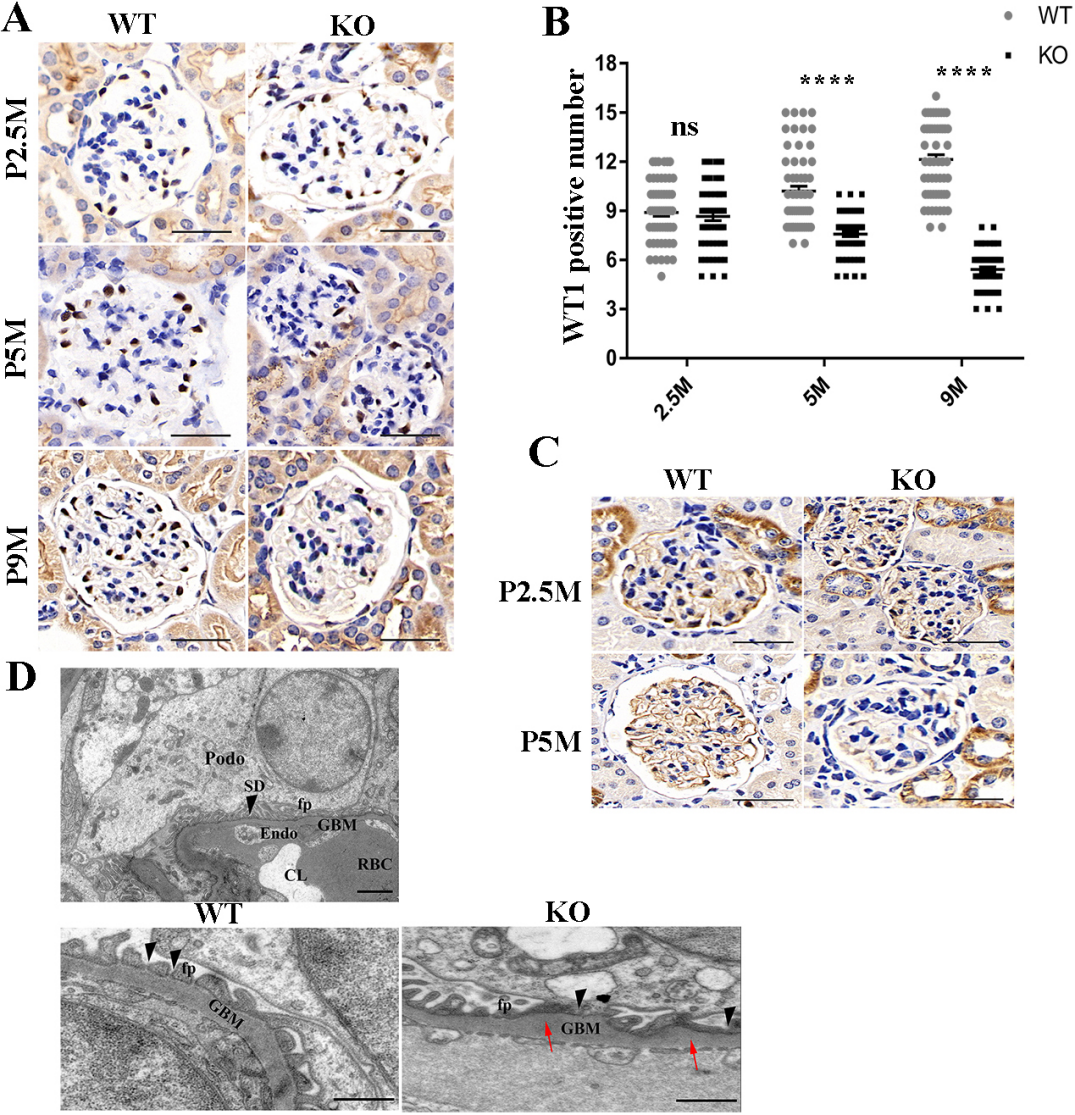
***Tmem30a* deficiency impaired podocyte survival and function.** (A) Immunohistochemical staining of kidney sections revealed that the state of the glomeruli in *Tmem30a* KO mice is comparable to that in WT mice at 2.5 months of age. However, the number of WT1-positive cells in glomeruli dramatically decreased after 5 months in the KO mice compared with the WT littermates, indicating podocyte degeneration in the *Tmem30a* KO mice. (B) Quantification of WT1-positive cells in the glomeruli of both WT and KO mice. *n*=3, 20 glomeruli were randomly selected from each sample. Values represent the mean±s.e.m. ns, not significant; ****P<0.0001, by Mann–Whitney *U* test. (C) Immunohistochemical staining of paraffin-embedded kidney sections from *Tmem30a* WT and KO mice for synaptopodin revealed the loss of synaptopodin by 5 months of age. Positive staining for synaptopodin was hard to detect at 5 months, indicating podocyte loss. (D) Transmission electron microscopy images of glomeruli in *Tmem30a* WT and KO mice at 5 months. The upper panel shows the normal glomerular filtration barrier and slit diaphragm (SD) formed between the adjacent foot processes (fp). Compared with WT mice, KO mice exhibited increasing glomeruli base membrane (GBM) (red arrows), fusion of foot processes and lack of slit diaphragms (black arrowheads). CL, capillary lumen; Endo, endothelium; Podo, podocyte; RBC, red blood cell. Scale bars: 50 μm (A,C); 2 μm (D, upper panel); 500 nm (D, lower panels).

*Tmem30a* deletion results in albuminuria, implying podocyte injury and loss in *Tmem30a* KO mice. We reasoned that this mouse model should allow us to address the question about the role of *Tmem30a* in the glomerular filtration barrier and progression of nephrotic syndrome. We next examined whether *Tmem30a* is required for the survival of podocytes. Paraffin sections from both *Tmem30a* KO mice and WT mice at 2.5, 5 and 9 months of age were subjected to immunostaining for Wilms' tumour-1 (WT1) and synaptopodin, which are two representative markers of differentiated podocytes. WT1 is the nuclear marker of differentiated podocytes used to assess the state of mature podocytes. In the kidney of *Tmem30a* KO mice, the number of WT1-positive cells in glomeruli was comparable to that in littermate controls at the age of 2.5 months; however, it was dramatically decreased by 5 months of age in a pattern consistent with the severity of diffuse glomerulosclerosis, indicating a loss of podocytes (Fig. 4A,B). Synaptopodin is an actin-associated protein that may play a role in actin-based cell shape and motility (Abbate et al., 2006; Asanuma et al., 2005). Synaptopodin expression was also observed in the podocytes of WT mice but was hardly detectable in KO mice (Fig. 4C). The results of immunostaining for WT1 and synaptopodin confirmed the loss of mature podocytes in *Tmem30a* KO mice, indicating that *Tmem30a* plays an essential role in the survival and function of podocytes.

To further examine the role of *Tmem30a* in FP formation, the ultrastructure in WT and KO mice at 5 months of age was analysed by transmission electron microscopy (TEM) (Fig. 4D). *Tmem30a* WT mice showed normal podocyte, podocyte FP and GBM architecture (Fig. 4D, upper- and lower-left panels). In contrast, *Tmem30a* KO mice showed podocyte FP effacement, lack of a SD and increases in the GBM (Fig. 4D, lower-right panel), suggesting that *Tmem30a* deficiency causes impaired podocyte FP formation or imbalanced protein–protein interactions within the SD multiprotein complex, resulting in an impaired filtration barrier in the kidney.

### Loss of *Tmem30a* in podocytes causes endoplasmic reticulum (ER) stress

**Fig. 5.**
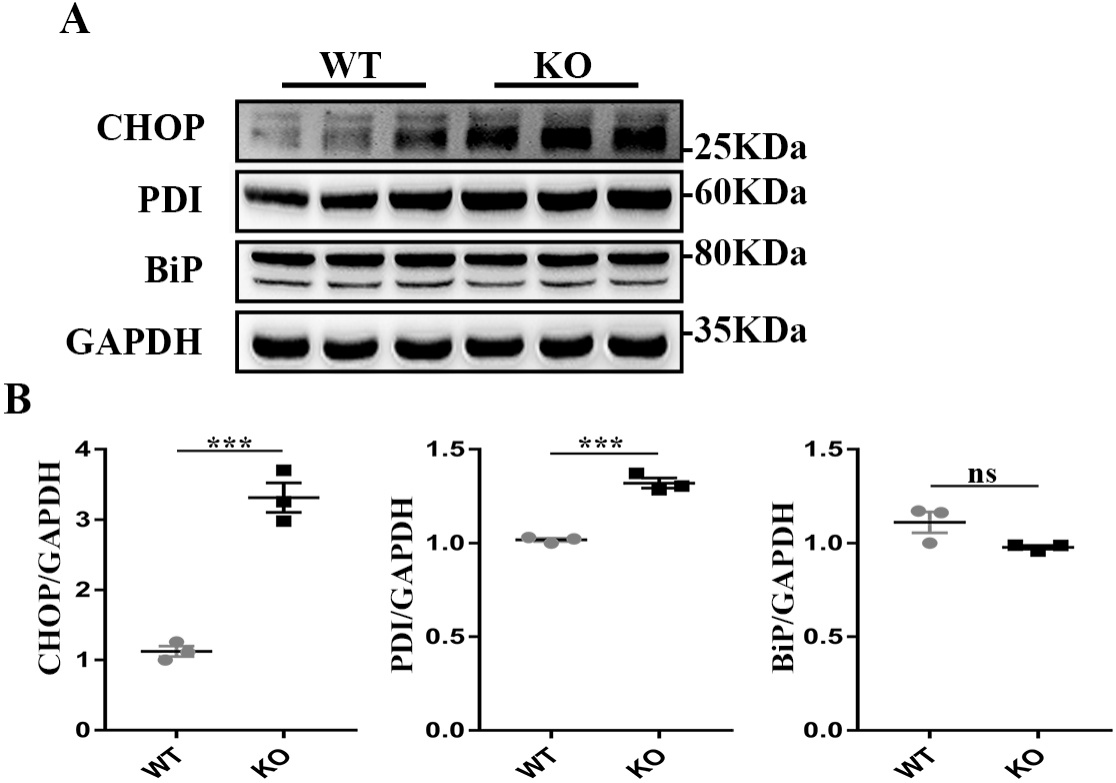
**Loss of *Tmem30a* causes endoplasmic reticulum stress in podocytes.** Western blot analysis of isolated glomeruli proteins in WT and *Tmem30a* KO mice at 5 months of age. (A) Western blotting was performed to detect the expression of CHOP, PDI and BiP, and GAPDH was probed as a loading control. (B-D) Quantitative analysis of blots. Sample size, *n*=3. Values represent the mean±s.e.m. ns, not significant; ***P<0.001, by unpaired Student's *t*-test.

A previous study suggested that the loss of *Tmem30a* in Purkinje cells induced ER stress and subsequent progressive degeneration of Purkinje cells, demonstrating the vital function of *Tmem30a* in intracellular trafficking (Yang et al., 2018). We speculated that podocyte injury and loss in *Tmem30a* KO mice might induce ER stress. Western blot analysis showed that the expression of ER stress-related proteins, including CHOP (also known as DDIT3) and PDI (also known as PADI2), was upregulated in *Tmem30a* KO mice compared with WT mice at 5 months of age, indicating the presence of ER stress in *Tmem30a* KO podocytes (Fig. 5).

### *Tmem30a* KO mice develop severe glomerulosclerosis

**Fig. 6.**
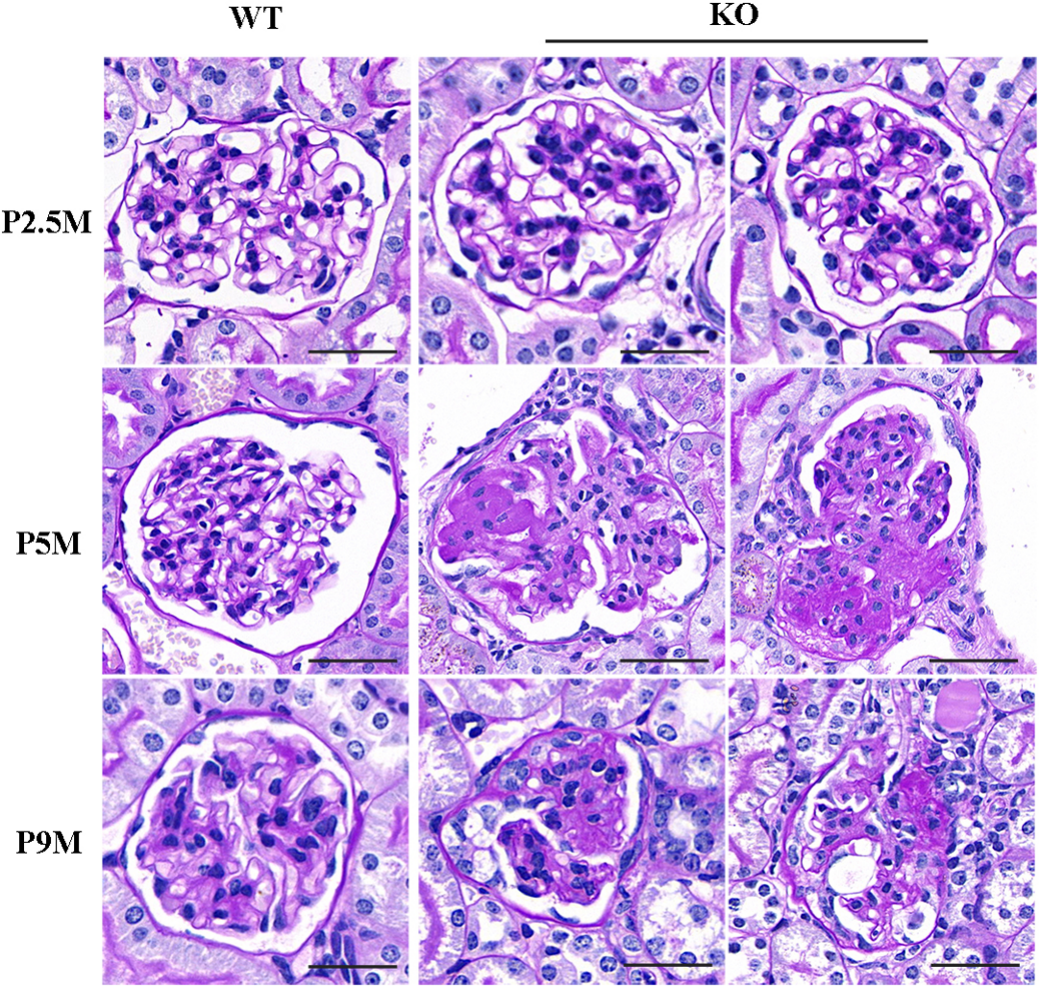
**Glomerular sclerosis in *Tmem30a* KO mice.** Representative light microscopy images of periodic acid–Schiff (PAS)-stained kidney samples from WT and KO mice showed slight segmental mesangial proliferation at 2.5 months. By the age of 5 months, glomeruli showed mesangial cell proliferation and increased extracellular matrix deposition with segmental glomerulosclerosis and (mild) adhesions to Bowman's capsule in *Tmem30a* KO mice. At 9 months of age, more glomeruli were damaged, and the glomeruli exhibited varying severities of pathological phenotypes as the disease progressed, such as mesangial cell proliferation and increased extracellular matrix deposition with segmental glomerulosclerosis (left panel of P9M KO) and adhesions to Bowman's capsule (right panel of P9M KO). *n*=3. Scale bars: 50 μm.

Kidney sections from both WT and KO mice at 2.5, 5 and 9 months of age were analysed by light microscopy to assess pathological changes. Periodic acid–Schiff (PAS) staining of kidney sections revealed normal nephrogenesis in *Tmem30a* KO mice, and the predominant renal changes were confirmed to be related to glomeruli (Fig. 6). The size of the kidneys in *Tmem30a* KO mice was generally the same as that of the kidneys in WT mice; deletion of *Tmem30a* in podocytes had no effect on kidney size (Fig. S2). Interestingly, histological analysis revealed grossly normal appearing glomeruli except for slight segmental mesangial proliferation in *Tmem30a* KO mice at 2.5 months of age. By 5 months, *Tmem30a* KO mice exhibited multiple pathological processes, including slight and severe mesangial hyperplasia, mesangial cell proliferation with ECM deposition and capsular synechia, and even glomerular sclerosis was visible throughout the renal cortex (Fig. 6, middle row, middle and right panels). By 9 months, prominent glomerular sclerosis was observed in *Tmem30a* KO mice (Fig. 6, lower row, middle and right panels). These data suggest that the kidney is undergoing a pathological process of FSGS, which also explains the absence of prenatal mortality.

PAS staining was also performed on the kidney sections of female and male mice at 8 months of age to observe whether sex had an effect on the glomerular pathological phenotype. The results showed that both female and male mice showed glomerulosclerosis (Fig. S1B).

## DISCUSSION

The β-subunit of the PS flippase TMEM30A is essential for generating and maintaining the asymmetrical distribution of phospholipids to ensure cellular signal transduction (Folmer et al., 2012; Huber et al., 2006; Paulusma et al., 2008; Kato et al., 2013). In this study, we reported decreased expression of TMEM30A in kidney sections of MCD and MN patients (Fig. 1), indicating the importance of TMEM30A in podocytes. As a β-subunit of PS flippase, TMEM30A might affect the transport of essential proteins for maintaining glomerular filtration barrier integrity in the kidney. By generating a podocyte-specific *Tmem30a* knockout mouse model, we demonstrated that *Tmem30a* indeed plays a vital role in maintaining glomerular filtration barrier integrity. Loss of *Tmem30a* leads to podocyte injury and loss, albuminuria, mesangial cell proliferation with mesangial matrix accumulation and eventually glomerulosclerosis as the disease progresses.

Podocyte injury and loss are now recognized as initiating factors leading to glomerulosclerosis in the progression of multiple variants of kidney diseases, such as DN, IgAN and FSGS (McConkey et al., 2004; Wiggins, 2007; Lemley et al., 2002; Petermann et al., 2004; Kihara et al., 1997). Podocytes are terminally differentiated cells that cannot repopulate after loss. Although a subpopulation of parietal epithelial cells can transform into podocytes, the capacity for regeneration appears to be limited and cannot compensate for the loss of podocytes (Vogelmann et al., 2003; Lasagni et al., 2010; Pippin et al., 2013; Zhang et al., 2013). Thus, podocyte injury and loss result in additional podocyte stress and, ultimately, glomerulosclerosis.

Given that *Tmem30a* plays a vital function in intercellular trafficking, we investigated the representative expression of the ER stress markers CHOP, PDI and BiP (also known as HSPA5) in isolated glomeruli. The results showed upregulated expression of CHOP and PDI in *Tmem30a* KO mice, implying induced ER stress in *Tmem30a* KO mice due to the loss of *Tmem30a* in podocytes. However, the expression level of BiP did not change significantly. These data suggested that the decreased *Tmem30a* affected ER stress by interfering protein modification and programmed cell death, not by affecting protein folding. Our previous study on *Tmem30a* in cerebellar Purkinje cells demonstrated that the loss of *Tmem30a* causes ER stress and eventually leads to degeneration of Purkinje cells (Yang et al., 2018). ER stress has already been confirmed in experimental models of primary human glomerulopathies, including MN, MCD, mesangial proliferative glomerulonephritis and FSGS (Cybulsky, 2010; Cybulsky et al., 2002, 2005; Inagi et al., 2008; Nakajo et al., 2007). These studies suggest that ER stress is involved in the pathogenesis of these renal diseases and that ER stress can aggravate proteinuria. Furthermore, increased expression of ER stress markers (such as ER chaperones or CHOP) in glomeruli has been detected in kidney biopsy samples of patients with MN, MCD, FSGS and proliferative glomerulonephritis (Cybulsky, 2013; Lindenmeyer et al., 2008). We evaluated the hallmark of the impaired integrity of the glomerular filtration barrier, albuminuria, and found that *Tmem30a* KO mice exhibited albuminuria at 2.5 months after birth, indicating impaired podocytes. Albuminuria became more severe in *Tmem30a* KO mice at 5 and 9 months after birth (Fig. 3). The deletion of *Tmem30a* in podocytes resulted in a compromised glomerular filtration barrier at 5 months of age. The decreased immunostaining of synaptopodin was due to podocyte injury. TEM analysis further identified podocyte injury in *Tmem30a* KO mice: the intercellular junction and cytoskeletal structure of the FPs were altered, and the cells exhibited an effaced phenotype, indicating podocyte injury (Fig. 4D). SD structures disappeared, and albuminuria developed.

Mounting evidence suggests that mesangial cells are activated in numerous glomerular diseases and undergo proliferation and phenotypic alterations in response to glomerular injury, allowing glomerular structural recovery (Hackl et al., 2013; Johnson et al., 1992). However, compensatory activity after injury leads to the proliferation of mesangial cells along with abnormal ECM deposition, which results in glomerular fibrosis or sclerosis (Samarakoon et al., 2013). PAS staining of samples from *Tmem30a* KO mice at 5 months showed multiple pathological processes; ∼12 out of ∼200 glomeruli in *Tmem30a* KO mice exhibited mesangial cell proliferation, increased ECM deposition and even segmental glomerulosclerosis. Furthermore, these pathological phenotypes became more severe and common at 9 months of age (Fig. 6). Considering the results on albuminuria levels, PAS staining and mature podocyte staining, *Tmem30a* KO mice were in the early stages of renal disease and began to develop phenotypes at 2.5 months of age. By the ages of 5 and 9 months, *Tmem30a* KO mice exhibited higher albuminuria levels and more glomeruli were affected. These results indicate that glomerular disease caused by the lack of *Tmem30a* in podocytes progresses rapidly. It is possible that filtered macromolecules become trapped in the mesangium, causing the over-reaction of mesangial cells and triggering an inflammatory response that plays a pivotal role in stimulating ECM synthesis, causing an imbalance between ECM synthesis and dissolution (Zhao, 2019; Santini et al., 2008). Persistent mesangial cell proliferation and ECM accumulation lead to glomerulosclerosis and end-stage rental failure (Schnaper et al., 2003).

In summary, our study reveals novel roles of *Tmem30a* in maintaining the integrity of the glomerular filtration barrier. The deletion of *Tmem30a* in podocytes resulted in podocyte degeneration, which led to a series of pathological phenotypic changes, including albuminuria, mesangial cell proliferation, mesangial matrix accumulation and glomerulosclerosis. One possibility is that *Tmem30a* deficiency causes defects in protein folding and transport in the ER, causing ER stress, which leads to podocyte injury and loss. As with our previous work in cerebellar Purkinje cells, loss of *Tmem30a* causes ER stress, which ultimately leads to the degeneration of Purkinje cells (Yang et al., 2018). Another possibility is that *Tmem30a* loss impairs lipid raft formation. Our previous study revealed that the deletion of *Tmem30a* in hematopoietic cells results in repaired lipid raft aggregation (Yang et al., 2019b). The SD is actually a lipid raft with a multiprotein complex, in which dynamic protein–protein interactions maintain the SD as the final form of selective filtration. This provides us with another unique perspective to understanding the mechanism of podocyte damage. Further investigation is necessary to elucidate the molecular signalling pathway in podocytes after the deletion of *Tmem30a*.

## MATERIALS AND METHODS

### Mouse model

All animal protocols were approved by the Ethics Committee of Sichuan Provincial People's Hospital. All animal experiments were performed according to the approved protocols and related guidelines. Mice were raised under a 12-h light/12-h dark cycle.

A conditional KO (cKO) allele carrying a floxed *Tmem30a* allele (*Tmem30a^loxP/loxP^*) has previously been described (Yang et al., 2018, 2019a; Zhang et al., 2017). To generate mice with *Tmem30a* deletion specifically in podocytes, *Tmem30a^loxP/loxP^* mice were crossed with transgenic mice expressing Cre recombinase under the control of the podocyte-specific podocin (NPHS2) promoter [podocin-Cre, B6.Cg-Tg(NPHS2-cre)295Lbh/J; The Jackson Laboratory, stock no. 008205; https://www.jax.org/strain/008205] (Moeller et al., 2003) to yield progeny with the genotype of *Tmem30a^loxP/+^*; N PHS2-Cre. Cre-positive heterozygous offspring were crossed with *Tmem30a^loxP/loxP^* mice to obtain *Tmem30a^loxP/loxP^*; NPHS2-Cre cKO mice. A tdTomato reporter was introduced to monitor the efficiency of Cre-mediated deletion of the floxed exon (strain name B6.Cg-*Gt(ROSA)26Sor^tm14(CAG-tdTomato)Hze^*/J; The Jackson Laboratory, stock no. 007914; http://jaxmice.jax.org/strain/007914.html). The reporter contains a loxP-flanked STOP cassette that prevents transcription of the downstream CAG promoter-driven red fluorescent protein variant tdTomato. In the presence of Cre recombinase, the STOP cassette is removed from the Cre-expressing tissue(s) in reporter mice, and tdTomato will be expressed.

### Genotyping by PCR

Genomic DNA samples obtained from mouse tails were genotyped using PCR to screen for the floxed *Tmem30a* alleles using primers for *Tmem30a*-loxP2-F, 5′-ATTCCCCTCAAGATAGCTAC-3′, and *Tmem30a*- loxP2-R, 5′-AATGATCAACTGTAATTCCCC-3′. Podocin-Cre was genotyped using generic Cre primers: Cre-F, 5′-TGCCACGACCAAGTGACAGCAATG-3′, and Cre-R, 5′-ACCAGAGACGCAAATCCATCGCTC-3′. TdTomato mice were genotyped using the following primers provided by the JAX mouse service: oIMR9020, 5′-AAGGGAGCTGCAGTGGAGTA-3′; oIMR9021, 5′-CCGAAATCTGTGGGAAGTC-3′; oIMR9103, 5′-GGCATTAAAGCAGCGTATCC-3′; and oIMR9105, 5′-CTGTTCCTGTACGGCATGG-3′. The first cycle consisted of 95°C for 2 min, followed by 33 cycles of 94°C for 15 s, 58°C for 20 s and 72°C for 30 s.

### Urine analysis

Twenty-four-hour urine samples were collected using metabolic cages. Collected urine samples were centrifuged at 500 ***g*** for 5 min, and the supernatant was used for the quantitation of albumin and creatinine. Quantitation of urinary albumin and creatinine was carried out using mouse albumin-specific ELISA kits (Roche) and creatinine determination kits (Enzymatic Method) (Roche), respectively, following the manufacturer's instructions.

### Renal pathology

Mice were anaesthetized with a combination of ketamine (16 mg/kg body weight) and xylazine (80 mg/kg body weight) and perfused transcardially with ice-cold PBS, followed by 4% paraformaldehyde in 100 mM PBS (pH 7.4). The kidneys were harvested, fixed in 4% paraformaldehyde, dehydrated and embedded in paraffin or optimal cutting temperature (OCT) solution for cryosectioning by standard procedures. Sections (2 μm) to be used for light microscopy analysis were subjected to PAS staining and visualized with a light microscope (Nikon Eclipse Ti-sr).

### Patient recruitment and ethics statement

The patient study was approved by the institutional review board of the Sichuan Provincial People's Hospital in Chengdu, China. All experiments were carried out in accordance with the approved study protocol, and all clinical investigation was performed according to the principles expressed in the Declaration of Helsinki. All enrolled patients signed written informed consent forms. Kidney tissues from IgAN, DN, MCD and MN patients were collected during renal biopsy in the Nephrology Department of Sichuan Provincial People's Hospital, and adjacent normal renal tissues were collected from patients with renal tumours during nephrectomy in the Department of Urology at the same hospital. All human kidney tissues underwent routine renal pathological examination to confirm the diagnosis. These tissues were processed by standard procedures for cryosectioning and immunofluorescence staining, as described below.

### Immunohistochemistry and immunofluorescence

Paraffin-embedded murine kidney slides (2 μm) were deparaffinized following a standard protocol. After washing and blocking, the tissues were incubated with primary antibodies against WT1 (1:100; Servicebio, GB11382) and synaptopodin (1:100; ZEN BIO, 508484). The slides were then incubated with horseradish peroxidase (HRP)-labelled donkey anti-rabbit secondary antibodies. Nuclei were visualized using 4′,6-diamidino-2-phenylindole (DAPI) counterstaining. Glomerular WT1 was determined by counting positively immunostained nuclei in 30 glomerular profiles in each kidney section. Images were taken using an Axioplan-2 imaging microscope with the digital image-processing program AxioVision 4.3 (Zeiss, Thornwood, NY, USA).

Frozen mouse tissues were sectioned at 5 μm (CryoStar NX50 OP, Thermo Fisher Scientific, Germany). After blocking and permeabilization with 10% normal goat serum and 0.2% Triton X-100 in PBS at room temperature for 1 h, the cryosections were labelled with the following primary antibodies overnight at 4°C: anti-TMEM30A (1:50; mouse monoclonal antibody Cdc50-7F4, gift from Dr Robert Molday, University of British Columbia, Vancouver, BC, Canada) and anti-nephrin (1:100; Abcam, Cambridge, MA, USA). The sections were rinsed in PBS three times and incubated with Alexa Fluor 488- or Alexa Fluor 594-labelled goat anti-mouse (Bio-Rad, STAR132P, RRID: AB_2124272) or anti-rabbit IgG secondary antibodies (1:500; Bio-Rad, 5213-2504, RRID: AB_619 907), and then stained with DAPI at room temperature for 1 h. Images were captured on a laser scanning confocal microscope (LSM800, Zeiss).

Frozen human tissues were sectioned using a cryomicrotome (MEV, SLEE, Germany) at 4 μm. To observe the expression of TMEM30A, cryosections were stained with rabbit anti-human TMEM30A (1:100; Bioss, Beijing, China) overnight at 4°C followed by fluorescein isothiocyanate-conjugated goat anti-rabbit IgG (1:100; Gene Tech Company Limited, Shanghai, China) at 37°C for 30 min. Images were captured using an Olympus BX51 microscope (Tokyo, Japan). All exposure settings were kept the same. The fluorescence intensity was measured by manually outlining the perimeter of every glomerulus and semiquantifying the luminosity of the outlined regions using image analysis software (ImageJ, version 1.52p, National Institutes of Health, USA). A background correction was made for each glomerulus by subtracting the average intensity in non-stained regions (outlined manually) in the glomerulus.

### TEM

TEM was performed on kidney cortical tissue (HITACHI, HT7700). Kidneys obtained from WT and KO mice were cut into small pieces just after harvest and fixed in fixative solution [2.5% glutaraldehyde, 1.25% paraformaldehyde, and 0.003% picric acid in 0.1 M sodium cacodylate buffer (pH 7.4)] for 2 h at room temperature. The fixed kidney was washed with 0.1 M PBS, postfixed with 1% osmium tetroxide (OsO_4_) in 0.1 M PBS (pH 7.4), and washed in 0.1 M phosphate buffer (pH 7.4) three times. The fixed tissue was embedded in Epon 812 after dehydration via an ascending series of ethanol and acetone and incubated at 60°C for 48 h. Ultrathin sections (60 nm) were cut and stained with uranyl acetate and lead citrate.

### Isolation of glomeruli

The glomeruli were dissected using standard sieving technique (Stevens and Oltean, 2018). Briefly, kidney were mashed with a syringe plunger and then pushed through 425 µm (top), 250 µm, 175 µm, 125 µm, 100 µm and 70 µm (bottom) sieves with ice-cold mammalian Ringer's solution (Shyuanye Biotechnology, Shanghai, China L15O10G100158) with 1% bovine serum albumin (BSA; A8010, Solarbio, Beijing, China). The glomerular tissue retained by the 100 µm and 70 µm sieves was collected into a centrifuge tube with ice-cold mammalian Ringer's solution and 1% BSA. The tube was centrifuged at 1000 ***g*** for 10 min at 4°C, then the supernatant was removed, and the glomeruli frozen in liquid N_2_ before storing at −80°C for further protein and RNA extraction.

### Western blotting

Isolated glomerular proteins were extracted in RIPA lysis buffer (50 mM Tris-HCl, 150 mM NaCl, 1% Triton X-100, 0.5% sodium deoxycholate and 0.1% SDS, pH 7.4) supplemented with complete protease inhibitor cocktail (Roche). The protein concentration was determined with the Bicinchoninic Acid (BCA) Protein Assay (Thermo Fisher Scientific). SDS-PAGE and western blot analysis were performed with equal amounts of protein (15 μg), which were then transferred to polyvinylidene difluoride membranes (GE Healthcare, Chicago, IL, USA). After blocking with 8% non-fat dry milk in Tris-buffered saline with Tween 20 (TBST) for 2 h at room temperature, the blots were probed with primary antibodies against CHOP (1:1000; Cell Signaling Technology, Danvers, MA, USA), BiP (1:1000; Cell Signaling Technology) and PDI (1:2000; Cell Signaling Technology) in blocking solution overnight at 4°C, followed by incubation with anti-mouse or anti-rabbit HRP-conjugated secondary antibodies (1:5000; Cell Signaling Technology, Danvers, MA, USA). The samples were normalized with anti-GAPDH (1:5000; Proteintech, Wuhan, China) primary antibody, and the relative intensity of the blots was quantified using ImageJ software.

### Statistical analysis

Data are expressed as the mean±s.e.m. Data sets were tested for normally distributed data using the Shapiro–Wilk test. Statistical analysis was performed using unpaired Student's *t*-test (or Mann–Whitney *U* test for nonparametric data) for comparison between two groups or one-way ANOVA followed by Dunnett multiple comparison test for comparison of multiple groups. *P*<0.05 was considered statistically significant.

## Supplementary Material

Supplementary information
