# Supplementary material for: Loss of phosphatidylserine flippase β-subunit Tmem30a in podocytes leads to albuminuria and glomerulosclerosis
Source: Dis Model Mech. 2021 Jun 25;14(6):dmm048777. doi: 10.1242/dmm.048777 (PMC8246268; doi:10.1242/dmm.048777)
Supplement: Supplementary information [file dmm-14-048777-s1.pdf]

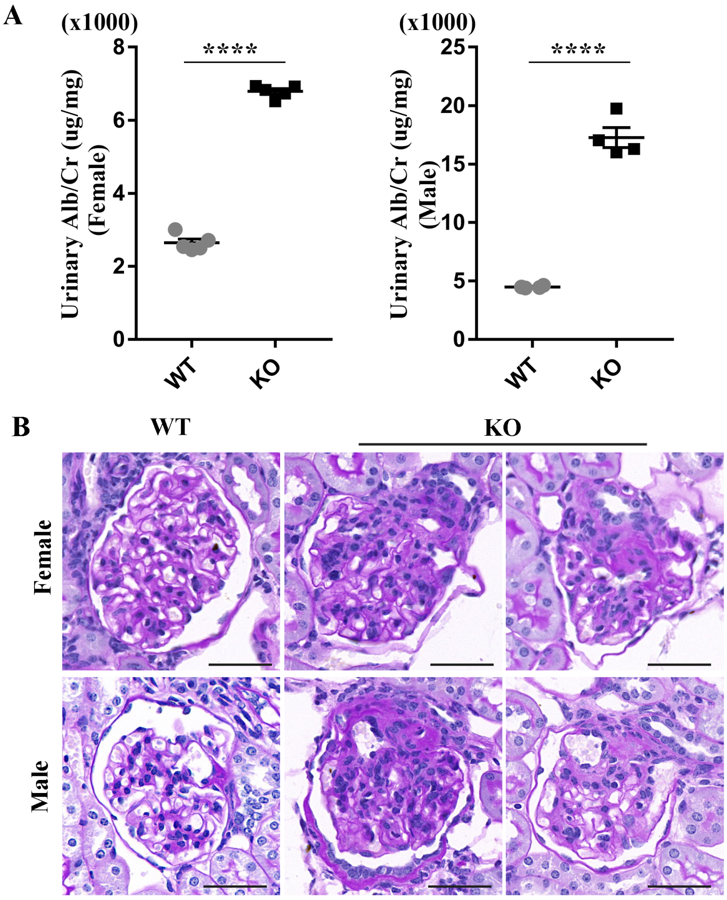


**Fig. S1. Both male and female *Tmem30a* knockout mice exhibit albuminuria and segmental glomerulosclerosis.** Albuminuria level and severity of glomerular lesions were assessed to exclude gender difference on glomerular disease progression in *Tmem30a* knockout mice at 8 months of age. (A) Albuminuria level was measured in female and male mice at same time point. Although the basal level of albuminuria of females were lower than those of males, both male and female *Tmem30a* KO mice showed a threefold increase in albuminuria levels compared to the same sex WT mice. Sample size, n=4. Values represent the mean ± SEM. ****$P<0.0001$ by Student's t-test. (B) Light microscopy images of PAS-stained kidney samples from female and male WT and KO mice. Both female and male Tmem30a KO mice showed segmental glomerulosclerosis. Sample size, n=3. Scale bar, 50 µm

Disease Models & Mechanisms • Supplementary information

**WT** **KO**

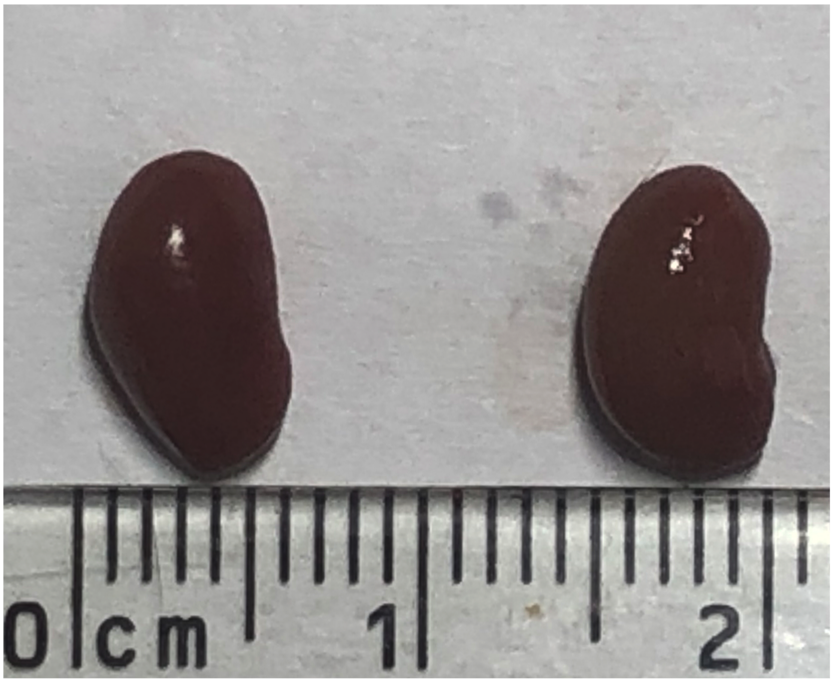


**Fig. S2. Deletion of *Tmem30a* in podocyte has no effect on kidney size**. There is no difference between WT and KO mice at 5 months.

Disease Models & Mechanisms • Supplementary information
